# Endoscopic Management of Post-Sleeve Gastrectomy Complications

**DOI:** 10.3390/jcm13072011

**Published:** 2024-03-29

**Authors:** Muaaz Masood, Donald E. Low, Shanley B. Deal, Richard A. Kozarek

**Affiliations:** 1 Division of Gastroenterology and Hepatology, Center for Digestive Health, Virginia Mason Franciscan Health, Seattle, WA 98101, USA; 2 Division of Thoracic Surgery, Center for Digestive Health, Virginia Mason Franciscan Health, Seattle, WA 98101, USA; donald.low@virginiamason.org; 3 Division of General and Bariatric Surgery, Center for Weight Management, Virginia Mason Franciscan Health, Seattle, WA 98101, USA; shanley.deal@virginiamason.org; 4 Center for Interventional Immunology, Benaroya Research Institute, Virginia Mason Franciscan Health, Seattle, WA 98101, USA

**Keywords:** endoscopy, endoscopic, management, sleeve, gastrectomy, gastric, sleeve, complications, leak, stenosis, fistula

## Abstract

Obesity is associated with several chronic conditions including diabetes, cardiovascular disease, and metabolic dysfunction-associated steatotic liver disease and malignancy. Bariatric surgery, most commonly Roux-en-Y gastric bypass and sleeve gastrectomy, is an effective treatment modality for obesity and can improve associated comorbidities. Over the last 20 years, there has been an increase in the rate of bariatric surgeries associated with the growing obesity epidemic. Sleeve gastrectomy is the most widely performed bariatric surgery currently, and while it serves as a durable option for some patients, it is important to note that several complications, including sleeve leak, stenosis, chronic fistula, gastrointestinal hemorrhage, and gastroesophageal reflux disease, may occur. Endoscopic methods to manage post-sleeve gastrectomy complications are often considered due to the risks associated with a reoperation, and endoscopy plays a significant role in the diagnosis and management of post-sleeve gastrectomy complications. We perform a detailed review of the current endoscopic management of post-sleeve gastrectomy complications.

## 1. Introduction

It has been reported that 38% of the world’s population are either overweight or obese [1]. Obesity, defined as a body-mass index (BMI) > 30 kg/m^2^, is associated with several chronic conditions including diabetes, cardiovascular disease, and metabolic dysfunction-associated steatotic liver disease and malignancy [2]. Bariatric surgery, most commonly Roux-en-Y gastric bypass (RYGB) and sleeve gastrectomy (SG), remains the most effective option for weight loss and the improvement of comorbidities [2,3,4]. With the growing obesity epidemic, there has been a rise in the rate of bariatric surgeries over the past twenty years [3,5]. SG surpassed RYGB in 2013 and has remained the most common bariatric surgery [5]. While SG is a durable option for some patients, it is important to note that several complications, including staple line leak, sleeve stenosis, fistula, gastrointestinal hemorrhage, and gastroesophageal reflux disease, may occur (Table 1) [6]. A multidisciplinary team, including gastroenterologists, bariatric surgeons, surgical endoscopists, interventional radiologists, and registered dieticians, is often called upon to manage post-SG complications. Endoscopic methods to manage SG complications are often considered given the risks associated with a reoperation, and endoscopy plays a significant role in the diagnosis and management of post-SG complications [7,8,9,10,11,12,13]. We perform a detailed review of the current endoscopic management of common post-SG complications.

## 2. Methods

A narrative review of the literature regarding complications following sleeve gastrectomy was conducted. PubMed computerized search and Google Scholar were utilized to identify articles with the following title or keywords: “sleeve gastrectomy”, “gastric sleeve”, “sleeve”, “complications”, “endoscopic”, “management”, “leak”, “fistula”, “stenosis”, “gastrointestinal hemorrhage”, and “gastroesophageal reflux disease”.

Articles were removed if they were not available in English or full text. Articles that were unrelated to the topic and duplicate articles were excluded. Systematic reviews, meta-analyses, and randomized controlled trials were assigned a high priority. A total of 132 articles were included in the final review. This article does not contain novel studies with human participants or animals conducted by any of the authors. The authors contributed original, anonymized radiographic and endoscopic images which serve to highlight the points outlined in the manuscript. The authors reviewed all articles within their discipline. The manuscript and images were independently reviewed and revised by all authors.

## 3. Discussion

### 3.1. Gastric Sleeve Leak

**Scheme 1:**
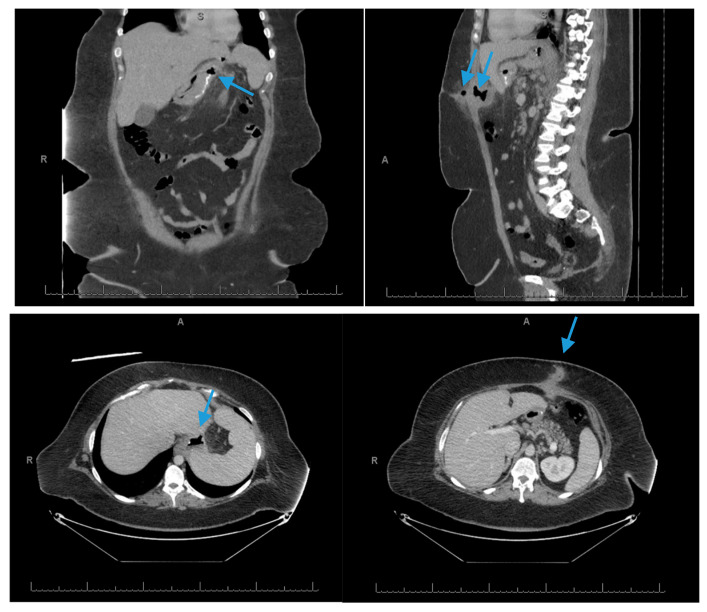
Lateral and transverse (**top left** and **top right**) views of a computed tomography scan of the abdomen and pelvis reveals air-fluid collections adjacent to the fundus of the stomach (3.3 × 3.0 cm) (arrow, **top left**), anterior to the stomach (2.2 × 4.6 cm) and in the epigastric region of the abdominal wall (6.2 × 3.0 cm) (arrows, **top right**), which likely represents abscesses in the setting of staple line dehiscence. Axial views of the computed tomography scan of the abdomen and pelvis (**bottom left** and **bottom right**) redemonstrate the somewhat ill-defined collection of gas and (arrows, **bottom left** and **bottom right**) fluid likely due to focal dehiscence and an abscess cavity which is the source of a gastrocutaneous fistula.

Gastric sleeve leak (GSL), or staple line leak, refers to the leak of gastrointestinal contents from the staple line into the abdominal cavity (Scheme 1) [14]. GSL most commonly occurs at the gastroesophageal junction, which has been attributed to mechanical factors (i.e., tension, thin gastric wall, increased pressure on the proximal sleeve in the setting of a distal sleeve stenosis) or tissue ischemia from the surgical ligation of the short gastric arteries [9,15]. GSL within the first 2 days postoperatively tends to be associated with mechanical etiologies (i.e., surgical technique), whereas GSL which presents >5–6 days postoperatively is more commonly associated with ischemic causes [10]. Recent data suggests that the incidence of GSL is 0.7% [16].

Csendes et al. and Burgos et al. proposed the classification of GSL according to timing, location, and severity [17,18]. Type I (subclinical) leaks are well-defined, without septic manifestations nor dissemination into the abdominal or pleural cavity. In contrast, Type II leaks (clinical) are characterized by septic manifestations and generalized dissemination into the abdominal or pleural cavity. Based on timing, leaks are classified as early, intermediate, and late if they occur 1–3 days, 4–7 days, and 8 or more days following surgery, respectively [19]. When GSLs are classified using clinical and radiologic data, Type A are microperforations without clinical or radiologic evidence of leak, Type B are leaks noted on radiographic studies without a clinical finding, and Type C are leaks with both radiological and clinical evidence [20]. Recently, a radiological classification system was proposed by Johari et al. which divided GSL into four groups, Class 1 through 4, which predicts the risk of complication severity and for salvage surgery and increased hospital stay based on CT features [21].

**Scheme 2:**
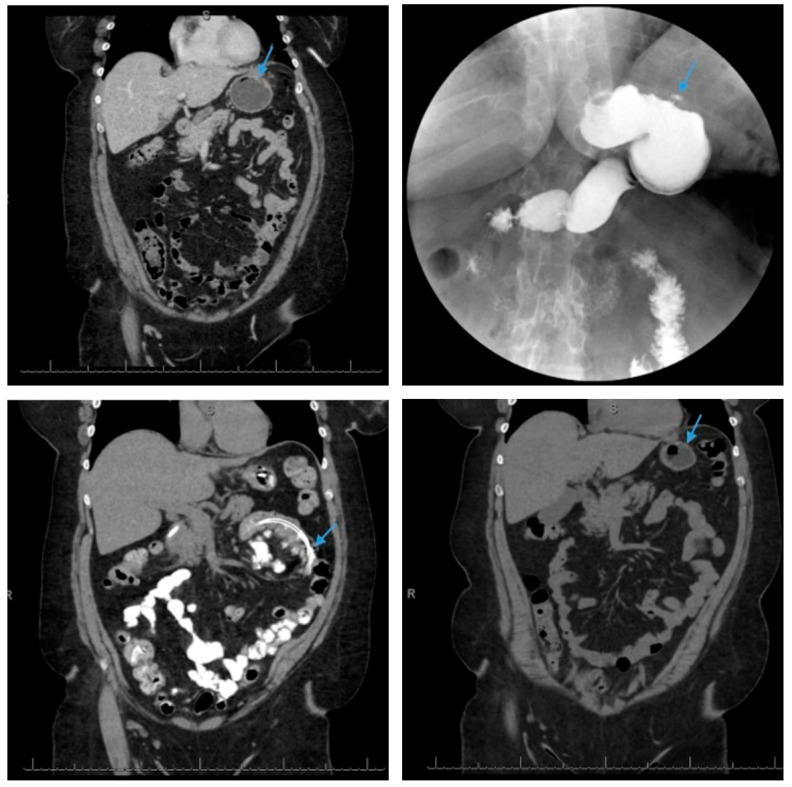
Coronal view of a computed tomography scan of the abdomen and pelvis reveals a small, contained leak (arrow, **top left**) along the greater curvature of the gastric body in a patient following sleeve gastrectomy. Upper gastrointestinal series demonstrates a small amount of extravasated contrast along the greater curvature of the gastric body with no free spill of extravasated contrast which is consistent with a small, contained perforation (arrow, **top right**) in a patient post-sleeve gastrectomy. The patient was treated with nothing per os and nasojejunal tube feeds (arrow, **bottom left**) for two weeks, with the resolution of sleeve leak and the removal of the nasojejunal tube (arrow, **bottom right**) as demonstrated on a follow-up coronal view of a computed tomography scan of the abdomen and pelvis.

GSL may be asymptomatic and diagnosed on routine postoperative imaging or manifest as sepsis, shock, abdominal pain, chest/shoulder pain, or dizziness [22]. As the clinical presentation of GSL can vary, a low threshold for the initiation of diagnostic work-up is recommended [23]. The diagnosis of GSL is typically made with upper gastrointestinal series and computed tomography scan [24,25]. Endoscopy may be helpful in establishing the diagnosis if imaging is equivocal [26]. Initial management should include broad spectrum intravenous antibiotics, proton pump inhibitors, fluid resuscitation, nothing per os, distal enteral tube feeding, and consultation with surgery and gastroenterology (Scheme 2). [15]. Patients who are hemodynamically unstable or have non-contained leaks generally require immediate surgical intervention [22]. Surgical approaches include early oversewing with or without omentoplasty and laparoscopic or open drain placement [24].

In a comprehensive review of 438 articles, the endoscopic treatment of GSL was demonstrated to be a highly effective approach in patients without a frank perforation [27]. There are several endoscopic techniques which have been utilized in GSL, including the use of covered self-expanding metal stents (CSEMS), endoscopic internal drainage with transgastric double-pigtail stents most often used in conjunction with the insertion of a percutaneous drain, over-the-scope clips (OTSC), endoscopic suturing, endoscopic tissue sealant application, endoscopic vacuum therapy, and endoscopic septotomy [26]. The choice of management is typically individualized to the patient’s clinical condition, the timing of the diagnosis, the location and size of the GSL [28]. The approach to management of patients with GSL should be multidisciplinary, involving advanced gastroenterology, bariatric surgery, and interventional radiology [29]. Additionally, most symptomatic patients with an acute staple line leak/GSL and an associated fluid collection will require the concomitant placement of a percutaneous drain placed surgically or by interventional radiology [22].

### 3.2. Endoscopic Stenting

**Scheme 3:**
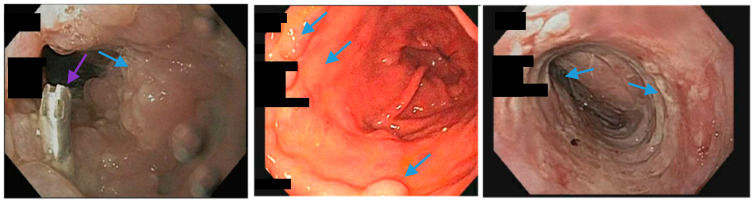
Upper endoscopy reveals an inflammatory mucosal reaction (blue arrows; **left**, **middle** and **right**) following the removal of a partially covered, esophageal self-expandable metal stent used for the treatment of a staple line leak in a patient with a sleeve gastrectomy. The patient reported severe regurgitation and chest pain associated with the stent. A through-the-scope clip (purple arrow, **left**) was used to anchor the removed stent.

The most common endoscopic technique to treat GSL has historically been the use of CSEMS which cover the leak orifice and can simultaneously treat distal sleeve stenosis if present. CSEMS are typically utilized when the GSL is <2 cm in size and located at the proximal or mid-portion of the sleeve [30]. Abscesses must be drained prior to the placement of CSEMS [30]. Chronic leaks seem to have the most favorable outcome with CSEMS [30]. Rogalski et al. explored the efficacy of self-expanding stents for the treatment of post-bariatric surgery leaks and fistulas [31]. Across 40 studies, successful leak or fistula closure with self-expanding stents was reported as 92% [31]. The stent typically remains in place for 6 to 8 weeks [10]. The endoscopic placement of a nasojejunal feeding tube may be needed to facilitate distal enteral nutrition in patients who undergo CSEMS placement. CSEMS may be poorly tolerated in some patients and can be associated with symptoms such as nausea, vomiting, GERD, and chest pain (Scheme 3). Patients with stents across the esophagogastric junction often require proton pump inhibitors. Many patients require CSEMS removal due to severe regurgitation and chest discomfort.

**Scheme 4:**
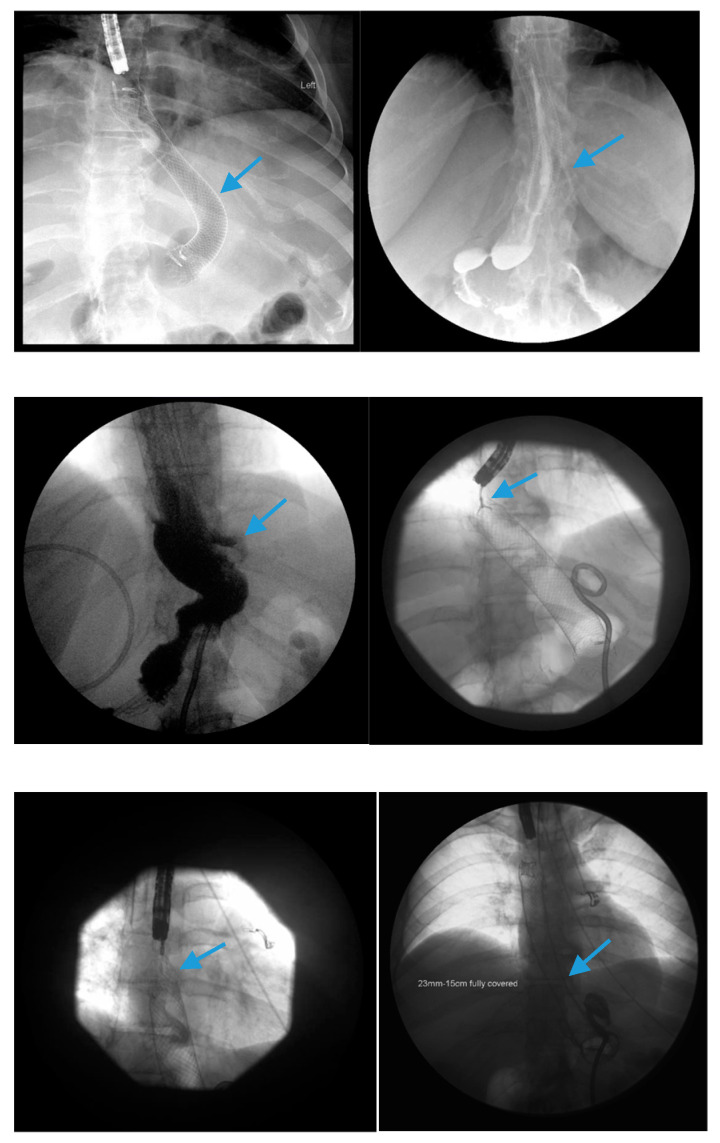
Fluoroscopic image demonstrates the placement of a 23 mm × 15 cm partially-covered esophageal self-expanding metal stent from the distal esophagus to the pylorus (arrow, **top left**) for the treatment of a leak in a patient following sleeve gastrectomy. Contrast injected endoscopically (arrows, **top right** and **middle left**) reveals a small, persistent sleeve leak. The endoscopic removal of the stent was subsequently attempted using a grasper (arrows, **middle right** and **bottom left**), but the stent removal was unsuccessful due to tissue ingrowth. A 23 mm × 15 cm, fully-covered, esophageal self-expanding metal stent was placed within the prior stent (arrow, **bottom right**) to facilitate removal after pressure necrosis of the ingrown tissue.

Stent migration remains a risk, and rates among studies vary from 22% to 59% [10,30,32,33]. Galloro et al. reported that the variable migration rate of CSEMS may be explained by the fact that the CSEMS commonly used for GSL are intended for use as esophageal stents, and thus CSEMS may not be adequately contained in the stomach [30]. CSEMS have a coating which prevents integration to the stomach wall but also facilitates stent migration [30]. Over-the-scope clips and endoscopic suturing have been utilized to anchor the proximal aspect of the CSEMS to the esophagus though the benefit of OTSC and endoscopic suturing on stent migration rate remains unclear [33]. Although partially covered SEMS have been associated with decreased stent migration due to the ingrowth of tissue over the partially covered aspect which serves to secure the stent, the removal of partially covered stents is often technically challenging, as illustrated in Scheme 4 [10]. Recently, bariatric-specific stents, i.e., Niti-S MEGA™ stent (Taewoong Medical Industries, Gimpo-si, Republic of Korea), Niti-S BETA™ stent (Taewoong Medical Industries, Gimpo-si, Republic of Korea), and Gastro-Seal™ stent (M.I. Tech, Pyeongtaek-si, Republic of Korea), have emerged which generally have a larger stent diameter and a longer stent length to suit patients with sleeve gastrectomy [32,34,35,36]. Despite a high leak closure rate with bariatric-specific stents, significant morbidity has been reported [37].

### 3.3. Endoscopic Internal Drainage with Transgastric Double-Pigtail Stents

**Scheme 5:**
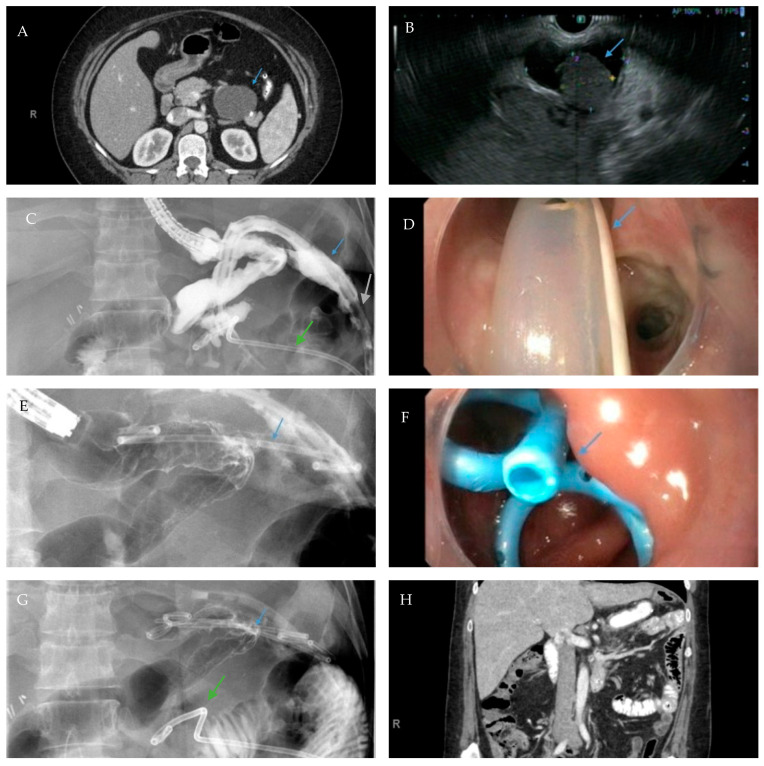
Axial view of a computed tomography scan of the abdomen and pelvis with contrast reveals a uniform, thin-walled, cystic lesion of the pancreas, which measures 90 mm × 52 mm × 48 mm, with peripheral calcifications with a BMI of 38 (arrow, **A**). Endoscopic ultrasound demonstrates an anechoic and hypoechoic lesion of the pancreatic body with two compartments, no septae or internal debris, and an associated mural nodule which was suggestive of a mucinous cystic neoplasm (arrow, **B**). The patient subsequently underwent a laparoscopic sleeve gastrectomy and a concurrent, open distal pancreatectomy which was complicated by a persistent pancreatic leak as well as a gastric sleeve leak (blue arrow, **C**) as demonstrated by contrast injected through a percutaneous drain (silver arrow, **C**). The percutaneous drain is redemonstrated on upper endoscopy (arrow, **D**). Note the additional percutaneous drain in the pancreatic bed for the postoperative leak (green arrows, **C**,**G**). Endoscopic internal drainage using transgastric double-pigtail stents was performed as demonstrated on fluoroscopic imaging (arrows, **E**,**G**) and endoscopy (arrow, **F**). After a prolonged course, the patient had a complete recovery, and a follow-up CT scan of the abdomen and pelvis confirmed the resolution of the leak and the removal of the double-pigtail stents and percutaneous drains (**H**).

Endoscopic internal drainage (EID), introduced in 2012, involves the use of transgastric double-pigtail stents to drain an abscess associated with a GSL which eventually results in the closure of the leak (Scheme 5) [38]. If the leak opening is large enough to allow for the safe passage of the endoscope, debridement of a chronic abscess cavity can be performed endoscopically prior to the placement of double-pigtail stents. The double-pigtail stents are typically exchanged every 6–8 weeks until leak closure. In a meta-analysis of 11 studies and 385 patients, the pooled proportion of successful leak closures with EID was reported as 84.71% [39]. In a more recent meta-analysis by Laopeamthong et al., leak closure rates of 91.6% were reported with EID across 10 studies [40]. The mean treatment duration for EID was 78.4 days [40]. EID is generally better tolerated than CSEMS and allows for the early resumption of an oral diet [41,42]. EID also does not expose patients to the risks associated with reoperation or percutaneous drainage (i.e., gastrocutaneous fistula) [43]. However, external drainage in conjunction with EID with double-pigtail stents is still used in individuals with a large fluid collection or superimposed sepsis. Some bariatric centers consider EID as the primary modality for the management of GSL especially for subacute leaks in hemodynamically stable patients [44].

### 3.4. Over-the-Scope Clips

**Figure 1:**
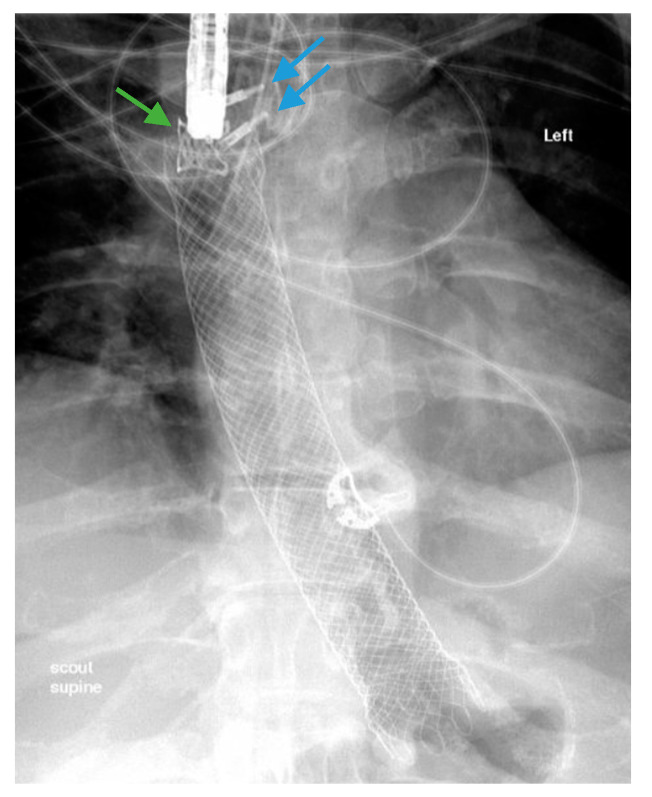
Upper gastrointestinal series reveals a 23 mm × 15 cm fully-covered esophageal self-expanding metal stent from the distal esophagus to the mid portion of the sleeve anchored by two through-the-scope clips (blue arrows) and one over-the-scope clip (green arrow) placed on the proximal edge of the stent.

**Scheme 6:**
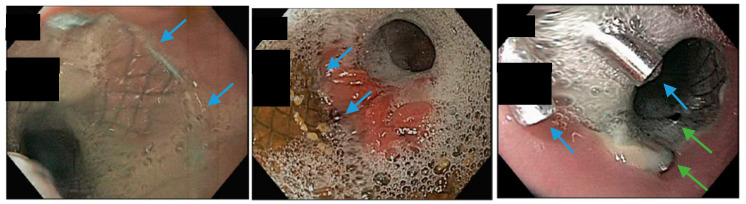
Upper endoscopy reveals the proximal (blue arrows, **left**) and distal edge (blue arrows, **middle**) of a fully-covered esophageal self-expanding metal stent anchored by two through-the-scope clips (blue arrows, **right**) and an over-the-scope clip (green arrows, **right**). Note copious saliva collected around the non-peristaltic prosthesis.

The over-the-scope clip system (Ovesco Endoscopy, Tübingen, Germany) has also been used for GSL closure [10,33]. The defect must be easy to access endoscopically and small, <10 mm in size [45]. The tissue at the site of the leak is often compromised due to ischemia or infection which can pose a challenge for successful, long-term closure with the use of clips [26]. OTSC should be avoided with large defects and chronic fistulas due to difficulty with tissue approximation [46]. In a meta-analysis of 10 studies and 195 patients with post-SG leak/fistula, leak closure with OTSC was successful in 86.3% of patients [47]. However, the data are limited due to a small sample size, short-term follow-up and OTSC being utilized as a part of a multimodal treatment approach [47]. In a more recent meta-analysis, the success rate for clipping alone was 67.1% with complications which included migration, sleeve stenosis, and tear [31]. The extent of the tear, i.e., mucosal, submucosal, or transmural, was unclear in the meta-analysis. OTSC are often used as an adjunct to other modalities for GSL closure (Figure 1 and Scheme 6) [48].

### 3.5. Endoscopic Suturing

**Figure 2:**
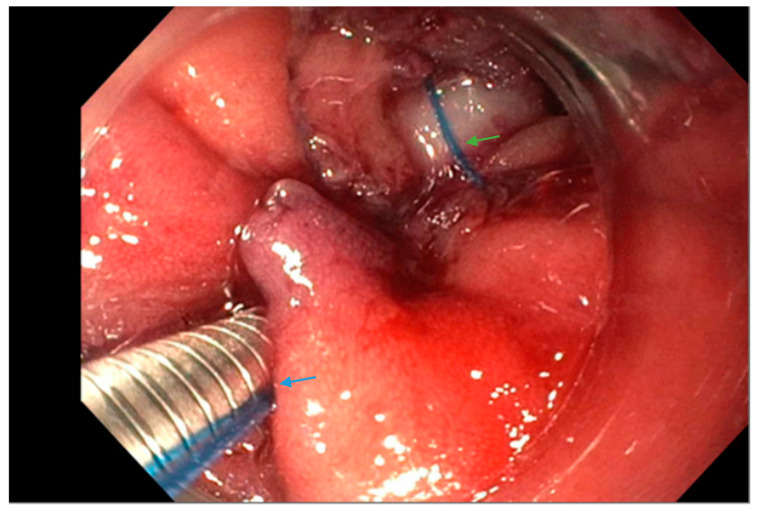
Upper endoscopy reveals the use of a suturing system (arrow, blue) with a suture in place (arrow, green) at the leak site in a patient following sleeve gastrectomy.

Endoscopic suturing involves the full-thickness closure of the leak using a suturing system, i.e., OverStitch (Apollo Endosurgery, Austin, TX, USA) (Figure 2) [49]. While endoscopic suturing can be effective when applied to healthy tissue, the quality of the tissue around a leak site is often poor due to ischemia or infection, which makes endoscopic suturing less effective particularly for leaks in which diagnosis is delayed [50]. When applied to friable, ischemic tissue around a leak site, endoscopic suturing may also lead to further tissue damage or an enlargement of the defect. Data regarding endoscopic suturing for full-thickness closure of sleeve leaks and fistulas are limited. While the technical success rate reported is high, the clinical success rate of endoscopic suturing was the lowest with anastomotic leak closure (27%) in a multicenter study of 122 patients [51]. In contrast, the clinical success rates for endoscopic suturing in the study were relatively higher with stent anchorage (91.4%), perforation (93%), and fistulas (80%) [51]. In a multicenter study of 56 patients with gastrointestinal fistulas, Mukewar et al. reported that all cases were technically successful. Persistent fistula closure was noted in 13 of 56 patients (23%), whereas 17 patients underwent additional endoscopic procedures of which four subsequently had successful closure [52]. A small study of six patients explored endoscopic full-thickness resection followed by purse-string sutures for post-LSG fistula [53]. A success rate of 83% was reported with no reported complications [53]. It is important to note that endoscopic suturing is often combined with other modalities for GSL closure, which may improve overall efficacy. Further studies are warranted to delineate the role of endoscopic suturing in the treatment of sleeve leaks.

### 3.6. Endoscopic Tissue Sealant Application

Tissue sealants, which include fibrin glue and cyanoacrylate, have been utilized for the treatment of post-SG leaks. Fibrin not only serves to seal the defect, but it also promotes wound healing through fibroblast proliferation and neovascularization. Fibrin glue is easily applied endoscopically, although multiple sessions are generally required for successful closure. The success rate for fibrin glue has been reported to be 92.8–100% [31]. A retrospective study of 1000 patients concluded that fibrin glue is a reliable and useful tool for staple line reinforcement [54]. However, a meta-analysis of 9991 cases revealed that bioabsorbable material did not significantly impact the incidence of fistulas [55]. A more recent meta-analysis exploring the efficacy of fibrin glue in reducing complications following bariatric surgery did not reveal a significant difference in the reduction of leakage with fibrin glue [56]. Carandina et al. stated that the surgical application of fibrin glue prolongs operative time, while other studies did not indicate an impact on operative time [57]. The use of fibrin seems to be relatively safe, with minor complications (i.e., pain, fever) reported [58]. Fibrin should not be used as a monotherapy for post-SG leaks.

Cyanoacrylate has high adhesive and antibacterial properties [59]. Cyanoacrylate is more cost-effective than fibrin glue and has been shown to be efficacious [60]. However, pro-inflammatory effects have been documented [10]. Some studies reported favorable results with modified cyanoacrylate, Glubran^®^ 2 (GEM Srl, Viareggio, Lucca, Italy), associated with chemical omentopexy [61,62,63]. Intraoperative reinforcement with tissue sealants at the time of the SG has been explored, and some authors conclude a decreased risk of complications including leaks [64,65]. Further studies are warranted to define the role of fibrin glue and cyanoacrylate in the management and prevention of sleeve leaks.

### 3.7. Endoscopic Vacuum Therapy

Endoscopic vacuum therapy refers to the use of a wound vac sponge which is endoscopically applied to the leak site. The wound vac sponge is attached to a nasogastric tube, and continuous suction is applied to assist in the removal of fluid, pus, and liquified necrosis from the area of disruption. The sponge is often placed extraluminally into the cavity and facilitates leak healing through the formation of granulation tissue. EVT is used for contained leaks and may be poorly tolerated in some patients. Multiple endoscopic procedures are almost always required as the wound vac sponge is typically exchanged every 3 to 7 days. In addition, a percutaneous endoscopic jejunostomy tube or a nasojejunal feeding tube for distal enteral feeding is usually required while the sponge is in place. EVT has been documented to be safe and efficacious [66,67,68,69,70]. The leak closure rate for EVT was reported to be 91.6% according to a recent meta-analysis [40]. Intriago et al. reported clinical success rates of 87.2% [71]. EVT may also have a role in refractory cases of sleeve leak [26].

### 3.8. Endoscopic Septotomy

Endoscopic septotomy is primarily used to drain mature leaks which contain a fibrous septum between the leak cavity and the gastric lumen. Endoscopic electrosurgical knives, through-the-scope scissors, or argon plasma coagulation may be used to perform the septotomy. Multiple sessions are generally required. The clinical success rate for endosopic septotomy has been reported to be 80–100% based on several studies [46,72,73,74]. Concomitant sleeve dilation has been recommended as it reduces intragastric pressure, allows a favorable pressure gradient for drainage of the associated abscess cavity into the distal sleeve, and facilitates gastric emptying [46,75,76].

### 3.9. Sleeve Leak Prevention

Several techniques to prevent GSL, including gentle handling of tissues, facilitating tissue fluid drainage prior to stapling, oversewing the staple line, and staple line reinforcement, have been reported to minimize leak [24,77,78,79]. Some surgeons perform an intraoperative methylene blue test or intraoperative endoscopy to test for GSL, while others use a large orogastric tube for sizing the sleeve and for a leak test [12,24]. The VisiG^®^ system (Boehringer Laboratories, LLC, Phoenixville, PA, USA) is commonly used as a sleeve calibration device and can also test for a leak. The use of tissue sealants, such as fibrin and cyanoacrylate, at the time of the sleeve gastrectomy have also been utilized. Additional studies have revealed that the use of a 40-French or larger bougie was associated with a decreased risk of GSL without significantly impacting weight loss [55,80].

### 3.10. Gastric Sleeve Stenosis

**Scheme 7:**
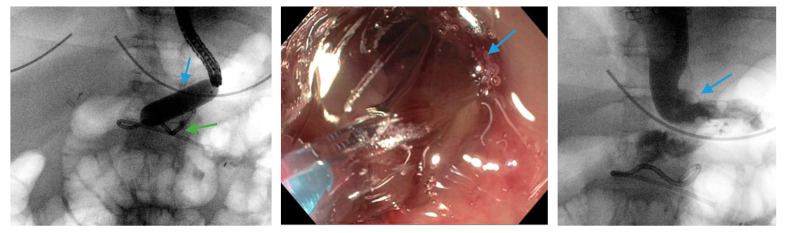
Gastric sleeve stenosis in the patient depicted in Scheme 5 was treated with through-the-scope balloon dilation to 20 mm as demonstrated on fluoroscopic image (arrow, **left**) and upper endoscopy (arrow, **middle**). Endoscopic contrast injection following dilation revealed improvement in the stenosis and flow of contrast (arrow, **right**). Note a small residual drain (green arrow, **left**).

The incidence of gastric sleeve stenosis (GSS) has been reported to be 0.5–3.5% [81]. GSS typically occurs at the level of the incisura and may be associated with a proximal sleeve leak due to increased pressure against the staple line and upstream dilation (Scheme 7) [82]. Patients may present with nausea, vomiting, dysphagia, or GERD. An upper gastrointestinal series is helpful in establishing the diagnosis and may reveal stenosis, upstream dilation, and contrast reflux. Endoscopy can reveal narrowing and angulation, though the endoscopist must be familiar with the condition. Contrast injection and fluoroscopic imaging are often utilized with endoscopy to assist in the diagnosis of GSS and for evaluation of post-treatment changes. In one study, endoscopy revealed sleeve stenosis in eight of 12 patients with sleeve stenosis on barium studies compared to one of five patients without stenosis on barium studies [83]. Levy et al. stated that barium studies allow for a direct visualization of the length and width of the sleeve and produce physiological distension [83]. In contrast, endoscopic evaluation assesses the sleeve lumen only and may result in artificial distension from insufflation which can mask areas of stenosis [83]. The study recommended the empiric endoscopic dilation of the aspect of the sleeve which is narrowed on a barium study in symptomatic patients [83]. However, the study was limited by its small sample size and retrospective nature [83].

### 3.11. Endoscopic Balloon Dilation

**Scheme 8:**
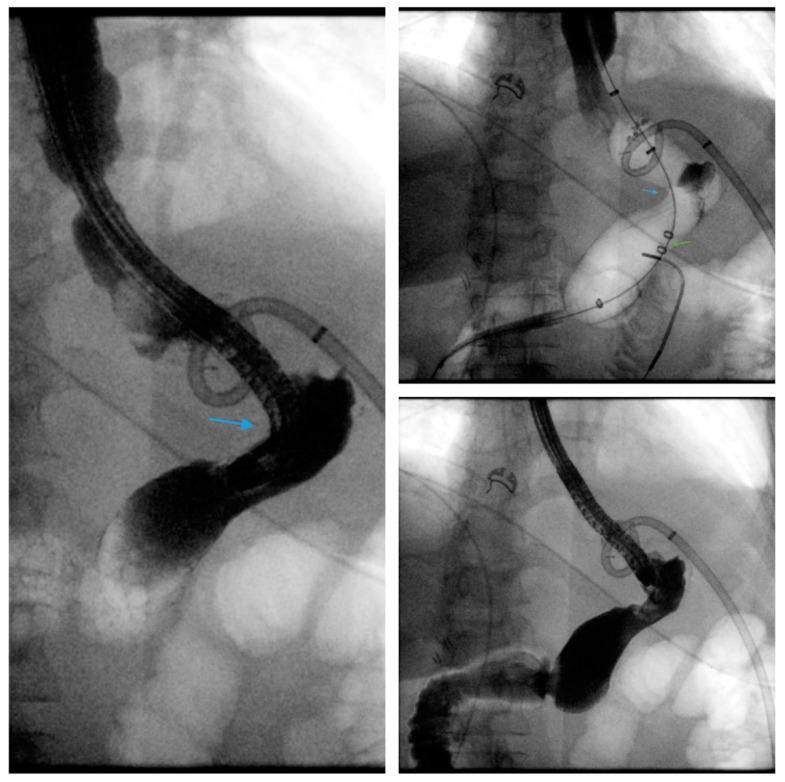
Contrast injected endoscopically reveals a sharp angulation and stenosis of the sleeve in a patient following sleeve gastrectomy (blue arrow, **left** and **top right**). A pneumatic balloon dilation (green arrow, **top right**) was performed to 35 mm. Endoscopic contrast injection reveals no post-dilation leak of contrast (**bottom right**).

GSS can occur if a narrow calibration tube is used to form the sleeve. Oversewing of the staple line or an abnormal rotation or kinking of the sleeve can also contribute to GSS. Endoscopic balloon dilation (EBD) has been reported to be safe and efficacious in the management of GSS [84,85,86]. A recent meta-analysis revealed an overall success rate of 76% and an average of 1.8 dilations per patient [48]. The success rate for EBD was higher in proximal GSS compared to distal GSS (90% vs. 70%) [48]. Some studies in the meta-analysis used through-the-scope continuous radial expansion balloons, while others used pneumatic balloons for achalasia or a combination of the two balloons. The optimal balloon type, the balloon size, and the number of dilations required remain unclear [49]. One approach is to start with a 20 mm continuous radial expansion balloon and, if stenosis or symptoms persist, to step-up to a 30 mm pneumatic balloon for achalasia inflated to waist effacement [87,88]. Serial dilations can then be performed with an incremental increase in inflation pressures and balloon sizes to a maximum diameter of 40 mm as tolerated (Scheme 8). A concomitant dilation of the pylorus up to 20 mm is sometimes performed to assist with symptoms if the pylorus is contributing to distal obstruction. Complications of EBD include bleeding or perforation. Fully covered self-expanding metal stents (FCSEMS) may be used if EBD is unsuccessful [89,90].

### 3.12. Role of Endoluminal Functional Lumen Imaging Probe in the Diagnosis of Sleeve Stenosis

Yu et al. reported an investigational approach to the diagnosis of GSS using endoscopic criteria [91]. The ratio between the narrowest and widest gastric lumen diameters and the presence of pooled fluid was associated with the diagnosis of GSS using an endoluminal functional lumen imaging probe (EndoFLIP) and gastric lumen morphology [91]. Other studies revealed that EndoFLIP measurements correlated with the endoscopic assessment of GS as well as quantified severity and predicted response to endoscopic dilation [92,93]. Gretchen et al. used endoscopic impedance planimetry measurements to provide new benchmark values for the diagnosis and severity of GSS [94]. Nevertheless, further studies are needed to establish endoscopic criteria for the diagnosis of GSS.

### 3.13. Gastric per-Oral Endoscopic Myotomy and Endoscopic Intrapyloric Botulinum Toxin Injections for Gastric Sleeve Stenosis or Delayed Gastric Emptying

Gastric per-oral endoscopic myotomy (G-POEM), first described in 2013 by Khashab et al., is an endoscopic technique which involves submucosal tunneling to incise distal antral and pyloric musculature in gastroparesis [95]. While G-POEM is effective in the treatment of medically refractory gastroparesis, it has also been explored as a treatment option in patients with GSS [96,97,98]. In a study of 13 patients with GSS, G-POEM had a clinical success rate of 77%, and no major complications were reported [99]. Hu et al. reported the case of a 61-year-old female post-SG with delayed gastric emptying who underwent per-oral endoscopic pyloromyotomy with a reduction in her gastroparesis symptoms [100]. Additionally, intrapyloric Botulinum toxin injections have been used to treat delayed gastric emptying, including after esophagectomy and pylorus-preserving gastrectomy [101,102,103,104,105,106]. Data regarding the endoscopic use of Botulinum toxin injection of the pylorus in the treatment of stenosis or delayed gastric emptying associated with sleeve gastrectomy are sparse. Youssef et al. explored intraoperative pyloric Botulinum toxin injections at the time of SG in 115 patients and revealed a decreased rate of gastric sleeve leak compared to patients in whom injection was not performed [107]. Further studies are warranted to explore G-POEM and endoscopic intrapyloric botulinum toxin in the management of sleeve gastrectomy complications.

### 3.14. Gastrointestinal Bleeding

Gastrointestinal (GI) hemorrhage following bariatric surgery has been reported in up to 4% of cases and generally occurs in the early postoperative period [108]. The most common site of GI hemorrhage in SG is along the staple line, short gastric vessel pedicles, and trocar sites [109,110]. GI bleeding following bariatric surgery may manifest as hematemesis, melena, tachycardia, or a decrease in hemoglobin/hematocrit. The algorithm for the management of upper GI bleeding is recommended and includes the initial establishment of large bore intravenous lines for fluid resuscitation, transfusion of packed red blood cells, and proton pump inhibitors [110]. Endoscopy is often performed for both diagnostic and therapeutic purposes [111]. There should be a low threshold to perform endoscopy under general anesthesia with airway protection due to the challenges associated with post-bariatric anatomy [88]. Several endoscopic tools are available to control the source of bleeding, including thermocoagulation (i.e., bipolar cautery, heater probe, argon plasma coagulation), injection therapy (i.e., epinephrine, hypertonic saline, sclerosants, cyanoacrylate), and mechanical devices (i.e., through-the-scope and over-the-scope clips) [112]. Epinephrine injection should be used in conjunction with another injection therapy, i.e., sclerosant, thermal, or mechanical methods, rather than as monotherapy [113]. An overtube may be utilized to remove large clots in cases of massive hemorrhage [114]. Data regarding the endoscopic hemostasis of GI bleed in the post-SG population are limited. Epinephrine injection and heater probe cautery were the most common interventions in a retrospective study of 30 patients who developed upper GI bleed post-RYGB [115]. Endoscopic treatment was successful in all cases of bleeding, with 17% of cases requiring a second EGD for rebleeding [115]. In a recent American Gastroenterological Association (AGA) Clinical Practice Update, mechanical techniques (i.e., through-the-scope clips) were preferred over thermocoagulation for hemostasis in order to minimize the risk of leak at the site of treatment and allow for the early reinitiation of venous thromboembolism prophylaxis [88]. Hemostatic powders, over-the-scope clips, and endoscopic suturing were reported as alternatives to mechanical techniques [88]. If endoscopy does not reveal the source of the bleed, imaging, especially CT angiogram, may be helpful to localize the bleed and perform endovascular intervention [110]. Post-SG patients should be counseled regarding risk factors for the development of GI bleeding from peptic ulcer disease to include the use of non-steroidal anti-inflammatory drugs, alcohol, smoking, and Helicobacter pylori infection. Proton pump inhibitors or histamine-2 receptor antagonists are often prescribed postoperatively to prevent ulcers and reflux [116].

### 3.15. Gastroesophageal Reflux Disease

Data has revealed an association of sleeve gastrectomy with a worsening of pre-existing GERD and the development of de novo GERD [117,118,119,120,121,122,123]. GERD typically manifests as heartburn and regurgitation, although atypical symptoms (i.e., cough, laryngitis, asthma, or chest pain) may be present in some patients. Masood et al. details the current management and treatment paradigms of post-SG GERD in a recently published review [124]. Endoscopy may be performed as part of a diagnostic work-up, which can also include esophagram, esophageal manometry, and pH testing, for further anatomical and physiological evaluation in selected patients presenting with GERD. Endoscopic balloon dilation may be performed in patients with GERD associated with GSS. There may be a role for endoscopic surveillance for Barrett’s esophagus post-SG [122,125,126]. Additionally, endoscopic SG has been described as a safe and efficacious primary weight loss procedure or revisional procedure for weight recidivism after SG [127,128,129,130]. ESG has also been associated with lower rates of new-onset GERD compared to laparoscopic SG [131]. In the MERIT trial of 209 patients with class 1 or 2 obesity who were randomly assigned to ESG or control, the primary endpoint of the mean percentage excess weight loss was greater for the ESG group compared to the control group (49.2% vs. 3.2%, *p* < 0.0001) [132]. Eighty percent of patients in the ESG group were reported to have an improvement in one or more metabolic comorbidities [132]. Further studies are warranted to determine the effect of endoscopic SG on GERD.

It is important to note that the treatment approach for post-SG complications should be individualized to each patient for optimal outcomes. Some endoscopic treatments can be poorly tolerated, and patients may require significant counseling and psychosocial support. Multidisciplinary collaboration with advanced gastroenterologists, bariatric surgeons, interventional radiologists, and registered dieticians in conjunction with effective communication are paramount to a successful outcome [88].

## 4. Conclusions

As the rates of obesity and bariatric surgery, particularly sleeve gastrectomy, continue to rise, clinicians, including gastroenterologists, should be familiar with the role of endoscopy in the diagnosis and management of post-sleeve gastrectomy complications. Various endoscopic techniques to treat gastric sleeve leaks are available including stenting, endoscopic internal drainage with transgastric double-pig tail stents, through-the-scope/over-the-scope clips, suturing, tissue sealants, endoscopic vacuum therapy, and septotomy. Sleeve stenosis may be effectively treated with endoscopic balloon dilation using either through-the-scope continuous radial expansion balloons, pneumatic balloons, or a combination of the two. Further studies are warranted to determine the role of endoluminal functional lumen imaging probe, gastric lumen morphology, gastric per-oral endoscopic myotomy, and endoscopic intrapyloric Botulinum toxin injections in the management of gastric sleeve stenosis. Tools for the treatment of gastrointestinal hemorrhage post-SG include thermocoagulation, injection therapy, and mechanical devices. Mechanical devices are preferred over thermocoagulation.

Finally, with regard to gastroesophageal reflux disease following SG, upper endoscopy is often performed for a further evaluation of reflux symptoms and for balloon dilation in cases of gastroesophageal reflux disease associated with gastric sleeve stenosis. Additional studies are needed to explore the effect of endoscopic sleeve gastroplasty on gastroesophageal reflux disease. Multidisciplinary collaboration with the promotion of strong relationships between advanced gastroenterologists and bariatric surgeons in addition to an individualized treatment approach for patients are key to a successful outcome in the endoscopic management of post-sleeve gastrectomy complications.

## Figures and Tables

**Table 1:** List of complications associated with sleeve gastrectomy and the current endoscopic management of each complication.

Sleeve Gastrectomy Complication	Endoscopic Management
Gastric sleeve leak/acute staple line leak Chronic fistula	Endoscopic stenting Endoscopic internal drainage with transgastric double-pigtail stents Over-the-scope clips Endoscopic suturing Endoscopic tissue sealant application Endoscopic vacuum therapy Endoscopic septotomy
Sleeve stenosis	Endoscopic balloon dilation o Through-the-scope continuous radial expansion balloon dilation o Pneumatic (achalasia) dilation Role of endoluminal functional lumen imaging probe in diagnosis Gastric per-oral endoscopic myotomy Endoscopic intrapyloric Botulinum toxin injections
Gastrointestinal hemorrhage	Thermocoagulation o Bipolar cautery, heater probe, argon plasma coagulation Injection therapy o Epinephrine, hypertonic saline, sclerosants and cyanoacrylate Mechanical devices o Through-the-scope, over-the-scope clips
Gastroesophageal reflux disease	Upper endoscopy (often in addition to upper gastrointestinal series, esophageal manometry and pH testing) for further evaluation of reflux symptoms Treatment of underlying sleeve stenosis, if present Endoscopic surveillance for Barrett’s esophagus following sleeve gastrectomy Endoscopic sleeve gastroplasty

## Data Availability

Not applicable.
